# Supplementary material for: Modification of perpendicular magnetic anisotropy and domain wall velocity in Pt/Co/Pt by voltage-induced strain
Source: Sci Rep. 2015 Jan 21;5:7921. doi: 10.1038/srep07921 (PMC4300497; doi:10.1038/srep07921)
Supplement: Supplementary Information — Supplementary Info - Modification of perpendicular magnetic anisotropy and domain wall velocity in Pt/Co/Pt by voltage-induced strain [file srep07921-s1.pdf]

Supplementary Information

Modification of perpendicular magnetic anisotropy and domain wall velocity in Pt/Co/Pt by voltage-induced strain

[1]P. M. Shepley, [2]A. W. Rushforth, [2]M. Wang, [1]G. Burnell, [1]T. A. Moore

[1]School of Physics and Astronomy, University of Leeds, Leeds, LS2 9JT, United Kingdom, [2]School of Physics and Astronomy, University of Nottingham, Nottingham, NG7 2RD, United Kingdom.

I. Strain Calibration

Here we show how strain from biaxial transducers is used to produce out-of-plane strain in Pt/Co/Pt films and how the strain can be measured from the longitudinal resistance of the Pt/Co/Pt Hall bars.

Definitions of strain in biaxial transducers

Figure S1 shows how the sample changes when a voltage is applied to the transducer. A negative voltage produces a biaxial expansion of the piezoelectric transducer in the x-y plane. This expansion stretches the Pt/Co/Pt on glass bonded to the top of the transducer. By geometrical arguments, as the Pt/Co/Pt is stretched in the x-y plane, its thickness t must decrease. We define this case as an out-of-plane compressive strain.

A positive voltage applied to the transducer causes a biaxial compression in the x-y plane. The in-plane dimensions of the Pt/Co/Pt on glass are reduced, so the material must expand in the z-direction. We define this as an out-of-plane tensile strain.

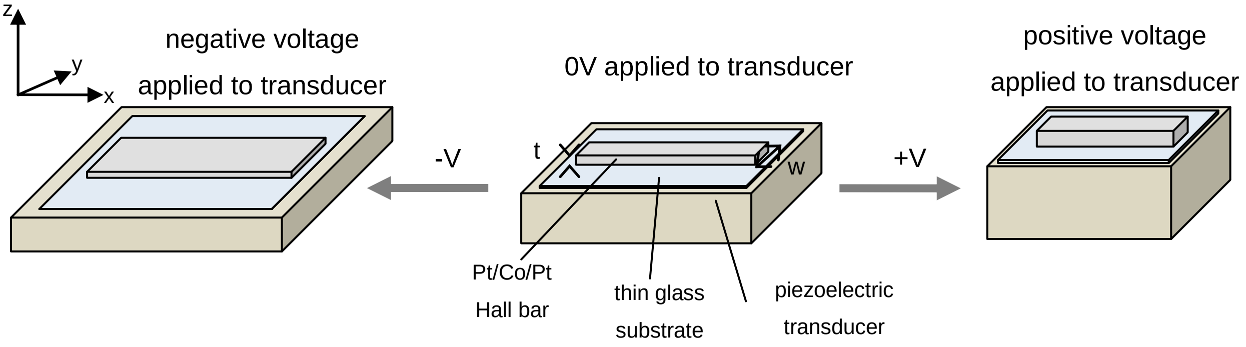

Figure S1 Schematic showing the effect of applying voltages to a biaxial piezoelectric transducer. A negative voltage causes tensile biaxial strain in the x-y plane (left hand side) and positive voltage causes compressive biaxial strain in the x-y plane (right hand side). The width w and thickness t of the Pt/Co/Pt Hall bar structure will also change.

Measurement of strain in Pt/Co/Pt thin films on biaxial transducers

The strain in a metal can be related to its change in resistance. By differentiating the resistance-resistivity relation $R = {\rho l}/{A}$, where R is the resistance of a wire, ρ is the resistivity, l is the length and A is the cross-sectional area, an expression relating the dimension change and resistance change can be obtained [S1].

$$\partial R = \frac{\rho}{A}\partial l - \frac{\rho l}{A^2}\partial A + \frac{l}{A}\partial \rho \qquad \text{(S1)}$$

$$\frac{\partial R}{R} = \frac{\partial l}{l} - \frac{\partial A}{A} + \frac{\partial \rho}{\rho} \qquad \text{(S2)}$$

$$\frac{\partial R}{R}\frac{1}{\varepsilon} = \frac{1}{\varepsilon}\left(\varepsilon - \frac{\partial A}{A}\right) + \frac{\partial \rho}{\rho}\frac{1}{\varepsilon} \qquad \text{(S3)}$$

The first term on the right of Equation S3 is a geometrical term. The second term is the strain coefficient of specific resistivity, which has been measured by Kuczynski [S2] as $\left(\frac{\partial \rho}{\rho}\frac{1}{\varepsilon}\right)_{Pt} = 2.60$ for bulk Pt and $\left(\frac{\partial \rho}{\rho}\frac{1}{\varepsilon}\right)_{Co} = 0.84$ for bulk Co. The mean value $\left(\frac{\partial \rho}{\rho}\frac{1}{\varepsilon}\right)_{Pt/Co} = 1.72$ has been used for this calculation. Thus the resistance-strain relation becomes

$$\varepsilon = \frac{\partial R}{R}\left(\frac{1}{geometrical + 1.72}\right) \qquad \text{(S4)}$$

For biaxial transducers the geometrical factor is found from

$$\frac{\partial A}{A} = \frac{\partial t}{t} + \frac{\partial w}{w} = \varepsilon_x - \frac{1}{\nu_z}\varepsilon_x \qquad \text{(S5)}$$

$$geometrical = \frac{1}{\varepsilon}\left(\varepsilon - \frac{\partial A}{A}\right) = \frac{1}{\varepsilon_x}\left(\varepsilon_x - \left(\varepsilon_x - \frac{\varepsilon_x}{\nu_z}\right)\right) = \frac{1}{\nu_z} \qquad \text{(S6)}$$

Here t is the thickness of the film or wire and w is the width (i.e. A=wt).

Giving an expression for the strain of

$$\varepsilon_x = \frac{\partial R}{R}\left(\frac{1}{\left(\frac{1}{0.36}\right) + 1.72}\right) = \frac{\partial R}{R}0.222 \tag{S7}$$

Figure S2 shows the strain measured for a Hall bar on a biaxial transducer. The strain in the y and z directions can be obtained from

$$-\frac{1}{\nu_z}\varepsilon_x = -\frac{1}{\nu_z}\varepsilon_y = \varepsilon_z \tag{S8}$$

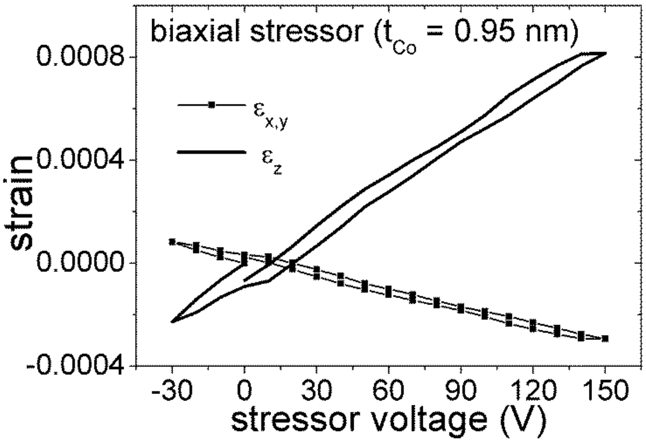

Figure S2 Strain in the x (along the Hall bar), y and z (out-of-plane) directions measured over a range of transducer voltages for a Pt/Co(0.95 nm)/Pt Hall bar on a biaxial transducer.

II. Deviation from coherent rotation of magnetization

Here we explain the behaviour of the magnetization during the extraordinary Hall effect (EHE) magnetic anisotropy measurements. The EHE signal is monitored while the magnetization is pulled from saturation out-of-plane to in-plane. If the magnetization were to rotate coherently then, following the Stoner-Wohlfarth model, magnetic free energy is given by

$$E = K_U \sin^2\theta - MH\cos(\theta - 90) \qquad \text{(S9)}$$

K_U is the uniaxial anisotropy, M is the magnitude of the magnetisation, θ is the angle of M with respect to the perpendicular direction and H is the magnetic field applied in the plane. Minimizing the energy with respect to θ gives

$$\theta = \sin^{-1}\left(\frac{MH}{2K_U}\right) \qquad \text{(S10)}$$

The EHE measurements give a signal that represents the perpendicular component of M

$$M_z = M\cos\theta = M\cos\left(\sin^{-1}\left(\frac{MH}{2K_U}\right)\right) = M\sqrt{1-\left(\frac{MH}{2K_U}\right)^2} \qquad \text{(S11)}$$

Therefore the out-of-plane component M_z has a parabolic shape with increasing in-plane field. Figure S3a shows the simulated coherent rotation along with an example of EHE data from a Pt/Co(1.0nm)/Pt sample. The data clearly deviate from this simple model at higher applied fields (>600 Oe in this case). We attribute this to the film breaking into domains. Kerr microscope measurements of Pt/Co(1.0nm)/Pt show contrast changes that suggest canted reverse domains nucleating then rotating until the film saturates. Figure S3 illustrates this process. Our analysis uses the first part of the EHE curve, where the magnetization rotates coherently, thus ensuring that the anisotropy field can be extracted.

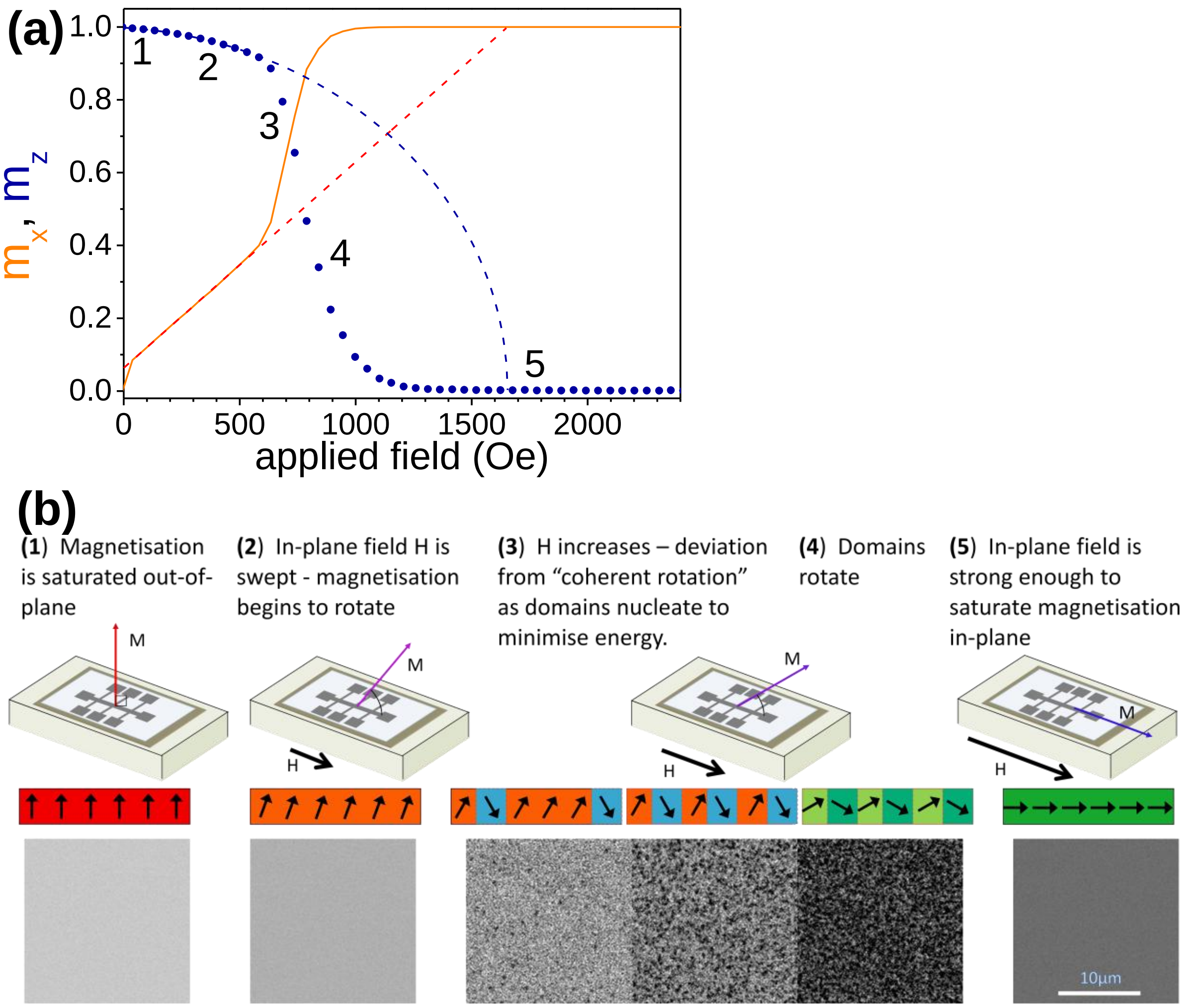

Figure S3. Rotation of magnetisation in Pt/Co(1.0nm)/Pt from out-of-plane to in-plane. (a) Normalised EHE data representing the out-of-plane magnetization component m_z (blue circles) and the in-plane component m_x (solid orange line) derived from this assuming that the total magnetization m = 1 throughout the measurement (so $m_x = \sqrt{1 - m_z^2}$). The dashed lines are a simulation of coherent moment rotation for the m_z (blue dash) and m_x (orange dash) components. The magnetic field is swept from 0 to -3000 Oe (b) Kerr images and cartoons of the magnetization rotation process, with numbered stages matching the numbered parts in (a).

References

[1] R.L. Parker, A. Krinsky. *Journal of Applied Physics*, **34**, 9 (1963)

[2] G.C. Kuczynski. *Physical Review*, **94**, 1 (1954)
